# Knowledge and Awareness of Cardiovascular Risk Factors Among Women With a History of Pregnancy Complications in Australia: A World Heart Federation Cross‐Sectional Study

**DOI:** 10.1111/ajo.70026

**Published:** 2025-04-01

**Authors:** Farnoosh Asghar Vahedi, Leila Gholizadeh, Marjan Khajehei

**Affiliations:** ^1^ School of Nursing and Midwifery Western Sydney University Sydney Australia; ^2^ School of Nursing and Midwifery, Faculty of Health University of Technology Sydney Sydney Australia; ^3^ Westmead Hospital Westmead Australia; ^4^ School of Medicine University of New South Wales Sydney Australia; ^5^ School of Medicine The University of Sydney Sydney Australia

**Keywords:** cardiovascular disease, cardiovascular risk, complications of pregnancy, gestational diabetes, hypertension disorders of pregnancy, long‐term cardiovascular health, pregnancy loss

## Abstract

**Background:**

Cardiovascular disease (CVD) is the leading cause of death worldwide, and women who experience complications during pregnancy, such as pregnancy loss (miscarriage and stillbirth), hypertensive disorders of pregnancy and gestational diabetes, have a significantly higher risk of future CVD. Increasing awareness of CVD risk among these women is crucial for effective engagement in risk reduction programs.

**Methods:**

A descriptive cross‐sectional survey using a non‐probability sampling method was adopted to explore the CVD knowledge and awareness of women with a history of complications of pregnancy. Distribution occurred from February to December 2021 via two tertiary hospitals in Australia and various groups in social media.

**Results:**

Of 364 survey responses received, a sample of 299 completed responses were considered for final analysis. The participants' mean knowledge score regarding CVD risk factors was 14.5 (SD ± 4.6; range 0–25). Among them, 50.2% had poor knowledge, 25.1% had moderate knowledge, and only 24.7% had good knowledge of CVD and its risk factors in women. Statistically significant differences in knowledge scores were observed based on ethnicity (*p* = 0.009) and education level (*p* = 0.007).

**Conclusion:**

This study revealed a significant gap in CVD knowledge among women with pregnancy complications, highlighting the need for targeted educational programs. Improving health literacy, particularly among high‐risk and lower socioeconomic groups, is crucial for reducing CVD incidence.

## 1 Introduction

Cardiovascular disease (CVD) is a major global health concern and the leading cause of death, accounting for approximately 30% of all deaths worldwide [1, 2]. In 2019, almost 17.9 million deaths were attributed to CVD globally, with a 21.1% increase from 2007 [2]. It is estimated that the number of CVD deaths is projected to rise globally from an estimated 18.9 million in 2020 to over 22.2 million in 2039 and 32.3 million in 2050 [3].

In the Australian population in 2022, CVD was the underlying cause of 24% of all deaths [4]. This indicates that CVD has been the second leading cause of death after cancer (which accounts for 27% of all deaths). Although the CVD mortality rate reached a notable low in 2020 at 158 per 100,000 population (during the first year of the pandemic), it ascended in both 2021 and 2022, rising by 2.3% and 2.1% respectively, after age adjustment [4].

There are significant discrepancies in CVD prevalence across different population groups, socioeconomic statuses, and geographical areas in Australia. For instance, from 2020 to 2022, the CVD death rate among people living in the lowest socioeconomic areas was 1.5 times greater than that of people living in the highest socioeconomic areas. Likewise, in 2021–2022, the hospitalisation rate for CVD among people living in remote and very remote areas was 1.3 times higher than that for individuals living in major cities [4] Moreover in 2018, CVD accounted for 10% of the total disease burden among First Nations people, with a higher proportion attributed to fatal burden than to non‐fatal burden (86% and 13%, respectively). The burden of disease from CVD was 2.4 times as high among First Nation people as among non‐Indigenous people [4].

CVD is the leading cause of death globally, and it is responsible for one in three deaths among women worldwide [5]. In Australia, nearly every hour, a woman dies from CVD, with postmenopausal women showing a higher prevalence than men of similar age (55% vs. 45%) [6]. There are several CVD risk factors which are shared between men and women, however, some risk factors affect women exclusively, known as female‐specific risk factors. These include complications of pregnancy, such as hypertensive disorders of pregnancy (HDP), gestational diabetes (GDM), and pregnancy loss [7, 8, 9]. Emerging evidence suggests a significant link between adverse pregnancy outcomes and an increased risk of CVD in women. For instance, a prospective longitudinal study in the United States by Tsulukidze et al. [10] found that a history of pregnancy loss, including natural losses and induced abortions, was associated with a 38% higher risk of CVD diagnosis (Odds Ratio (OR) 1.38). After adjusting for confounding factors, the risk remained elevated at 18% (adjusted OR 1.18). Similarly, a study in the United Kingdom reported that women with a history of stillbirth faced an increased risk of CVD (adjusted Hazerd Ratio (HR) 1.22; 95% Confidence Interval (CI) 1.01–1.46) and stroke (adjusted HR 1.44; 95% CI 1.12–1.85). Furthermore, pre‐eclampsia and gestational hypertension have been linked to a 2–4‐fold higher risk of long‐term cardiovascular complications, including heart failure and ischemic heart disease Oikonomou et al. [11]. Another UK‐based retrospective cohort study revealed that women with gestational diabetes mellitus (GDM) were over 2.5 times more likely to develop ischemic heart disease (Relative Ratio 2.78; 95% CI 1.37–5.66; *p* = 0.005) compared to those without GDM Daly et al. [12]. These findings emphasise the need to recognise adverse pregnancy outcomes as critical indicators of future cardiovascular health risks in women.

Enhancing awareness and education about these risks is critical, as most cases of CVD can be prevented with proper risk modification [13]. This study evaluates the knowledge and awareness of CVD risk in women with a history of complications of pregnancy.

## 2 Methods

### 2.1 Study Design

This was a descriptive cross‐sectional survey. We used a non‐probability sampling method, specifically a convenience sample to recruit participants in this study.

### 2.2 Ethical Approval

Ethical approval was obtained from the Sydney Local Health District Human Research Ethic Committee (approval number: X19‐0437 & 2019/ETH13440).

### 2.3 Sample Size

The sample size was calculated based on the results of previous studies, where knowledge of CVD risk was found to range from 10% to 44% [14]. Accordingly, we assumed that only 25% of the target population would have a reasonable general knowledge of CVD. Using the Select Statistical Services Population Proportion Sample Size Calculator, a sample size of 288 participants was determined to be required for the survey. To account for incomplete responses, a total of 299 women were recruited for the study, with a margin of error of 5% and a 95% confidence interval.

### 2.4 Study Population

Women who met the following inclusion criteria were invited to participate in the study: (a) aged 19 years or older; (b) proficient in speaking, reading, and understanding the English language; (c) able to provide informed consent; (d) having had a history of at least one pregnancy complication, including HDP, GDM, miscarriage, and stillbirth. Women with severe physical health issues or self‐reported mental and/or cognitive impairment were excluded from the study.

### 2.5 Study Setting

Women were recruited from several healthcare settings, including women's health ambulatory Clinics, antenatal clinics, early pregnancy clinics and postnatal wards at two major metropolitan public Hospitals in Sydney, Australia. To ensure maximum representativeness of the sample, the study Flyer was also posted on several facebook pages.

### 2.6 Data Collection Tool

We conducted a comprehensive review of the literature assessing CVD knowledge among various populations and groups, and consulted with experts in the field. As a result, a four‐section questionnaire was developed:

#### 2.6.1 Sociodemographic and Obstetric History

This section consisted of nine questions about participants' sociodemographic characteristics, including age, marital status, education, ethnicity and financial status, as well as their obstetric history, including the history of miscarriage, stillbirth, HDP and GDM.

#### 2.6.2 Cardiovascular Risk Factors

This section consisted of 17 questions about traditional CVD risk factors among participants and their engagement in risk‐reducing behaviours. This included questions about their history of high blood pressure, diabetes, and hyperlipidaemia, family history of CVD, BMI, physical inactivity, smoking status and whether they were aware of their risks or had regular medical assessments.

#### 2.6.3 Awareness of CVD Risks in Women

This section consisted of five questions on the awareness of participants about CVD among women.

#### 2.6.4 Knowledge of CVD Risk Factors

This section consisted of 22 questions on the knowledge of participants about CVD risk factors among women.

Responses to each question were ‘Yes’, ‘No’ and ‘I do not know’. The total knowledge score ranged from 0 to 22 and was calculated by summing up the correct answers to the questions, with higher scores indicating greater level of knowledge. Knowledge scores were categorised into three categories: poor, moderate, and good knowledge, using Bloom's cut‐off points: poor knowledge (a score ≤ 50% of correct responses), moderate knowledge (a score within 51%–69% of correct responses), and good knowledge (a score ≥ 70% of correct responses and above) [15].

The face and content validity of the developed questionnaires were established by a panel of eight experts (who were specialists on the topic) who also reviewed the test specifications and the selection of items. To assess the internal consistency of the questionnaires, we conducted a reliability test and the Cronbach's alpha coefficient was calculated to be 0.869.

### 2.7 Data Collection Procedure

The survey package made available both online on the Research Electronic Data Capture (REDCap, Vanderbilt University, Nashville, TN, USA) and in paper format, offering participants flexibility and increasing response rates.

Eligible women were recruited through selected hospitals, where the researcher explained the study, provided the participant information sheet and addressed questions. Interested participants signed a consent form to complete the paper survey or were given an online survey link.

Further to this, the survey link was posted on several Facebook pages, including ‘High Risk Pregnancy Support’, ‘High Risk Pregnancy Support Group’, ‘Gestational Diabetes Support & Meal Ideas’, ‘Rainbows After A Storm—Trying to conceive, Pregnancy and Babies After Loss’, and ‘High Risk Pregnancy Center’. After obtaining permission from the Facebook‐group administrators, the researcher posted the link to the study along with a copy of the participant information sheet on the selected pages. Posts included the participant information sheet, which noted that survey submission implied consent. The online survey remained open for 10 months, from February to December 2021.

### 2.8 Data Analysis

After data collection, data were exported from REDCap to Excel and then analysed using Statistical Package for Social Science, Advanced Statistics, Release 27.0 (IBM Corp. Released 2020. IBM SPSS Statistics for Windows, Version 27.0. Armonk, NY: IBM Corp). Descriptive statistics summarised sociodemographic data, pregnancy complications, and CVD risk factors. Frequency percentages, means, standard deviations (SD) and one‐way ANOVA were used to assess and compare participants' knowledge of CVD and its risk factors.

## 3 Results

### 3.1 Participant Sociodemographic Characteristics

Out of 364 survey responses received, 65 participants did not complete the entire survey and were excluded, resulting in a sample of 299 complete responses considered for final analysis. The mean age of the participants was 33.2 years (SD ± 5.2, range 19–49). The majority was married (78.3%) and described their financial condition as good (71.2%). More than half of the participants (58.5%) identified themselves as Australian, and 60% held a university degree. The sociodemographic characteristics of the participants are presented in Table 1.

**TABLE 1:** Participant sociodemographic characteristics (*n* = 299).

	Frequency	Percentage^a^
Age group		
< 25	13	4.3
25–35	190	63.5
> 35	96	32.1
Marital status		
Single/never married	17	5.7
Married	234	78.3
De facto	40	13.4
Divorced/separated	8	2.7
Ethnicity		
Australian including Australian Aboriginal/Torres Strait Islander	175	58.5
North African/Middle Eastern/Sub Saharan African	71	23.7
European/British/Irish	25	8.4
New Zealander	24	8.0
Southeast Asian/North Asian	4	1.3
Education		
Primary or secondary schooling	22	7.4
Certificate or diploma	97	32.4
Bachelor's or postgraduate degree	180	60.2
Financial situation		
Excellent	15	5.0
Good	213	71.2
Fair	65	21.7
Poor	6	2.0

^a^ Percentages may not add up to 100 due to rounding.

### 3.2 Participant CVD Risk Factors

Table 2 displays the CVD risk factors of participants. More than half of the participants (53.5%) were overweight or obese, 90.3% had moderate physical activity less than 150 min per week, and 39.8% had high blood pressure.

**TABLE 2:** Participant CVD risk factors (*n* = 299).

	Frequency	Percentage^a^
BMI		
Underweight (< 18.5 kg/m^2^)	3	1.0
Normal weight (18.5–24.9 kg/m^2^)	136	45.5
Overweight (25–29.9 kg/m^2^)	101	33.8
Obese (≥ 30 kg/m^2^)	59	19.7
Smoking status		
Current smoker	12	4.0
Physical activity		
Moderate physical activity at least 150 min per week	23	7.7
Moderate physical activity less than 150 min per week	270	90.3
Little or no physical activity	6	2.0
High blood pressure	119	39.8
Diabetes	70	23.4
Hyperlipidaemia	18	6.0
Family history of CVD	34	11.4

^a^ Percentages may not add up to 100 due to rounding.

A total of 43.1% of participants had at least one CVD risk factor, 25.8% had two CVD risk factors, and 14.4% had three or more CVD risk factors.

### 3.3 Participant Awareness of CVD Risk

The participants' knowledge and awareness of CVD is detailed in Table 3. A relatively small percentage of participants (24.4%) were aware that heart disease is the leading cause of death for women in Australia. Moreover, only 28% of respondents understood that women are more likely to die from heart disease than breast cancer. Nevertheless, most participants (62.2%) were aware that heart disease can manifest as asymptomatic or ‘silent’ in certain individuals. Regarding knowledge of heart‐attack signs and symptoms, 65.6% of participants either did not know or were uncertain that feelings of weakness, light‐headedness, or fainting could be indicative of a heart attack in women.

**TABLE 3:** Participant awareness of CVD risk in women (*n* = 299).

Statements	True	False	I don't know
	*n* (%)^a^	*n* (%)	*n* (%)
Heart disease is the number one killer of women in Australia	73 (24.4%)	58 (19.4%)	168 (56.2%)
Women are more likely to die from breast cancer than heart disease	89 (29.8%)	84 (28.1%)	126 (42.1%)
A woman would always know if she had a heart disease	52 (17.4%)	186 (62.2%)	61 (20.4%)
Heart disease is better defined as a short‐term illness than a chronic or long‐term illness	40 (13.4%)	198 (66.2%)	61 (20.4%)
Feeling weak, lightheaded, or faint are common symptoms of heart attack	103 (34.4%)	63 (21.1%)	133 (44.5%)

^a^ Percentages may not add up to 100 due to rounding; *n* = frequency.

### 3.4 Participant Knowledge of CVD Risk Factors in Women

Table 4 summarises the participants' level of knowledge in relation to their sociodemographic and clinical characteristics. The mean knowledge score of the participants regarding CVD risk factors was 14.5 (SD ± 4.6; range 0–25). Half of the participants (50.2%) displayed poor knowledge, one‐quarter (25.1%) exhibited moderate knowledge, and only 24.7% demonstrated good knowledge of CVD and its risk factors in women. When comparing the level of knowledge of participants based on their sociodemographic characteristics, statistically significant differences were noted in knowledge scores based on participants' ethnicity (*p* = 0.009) and education level (*p* = 0.007). Participants with Southeast Asian/North Asian backgrounds had the highest mean knowledge score (24.0 ± 3.8), while participants with Australian background had the lowest knowledge level (15.6 ± 5.3). Participants with a bachelor's or postgraduate degree had the highest level of knowledge compared to other groups (16.9 ± 5.5). Moreover, when the level of participant knowledge was compared based on their clinical characteristics, a significant difference in knowledge score was observed only in relation to participants' smoking status, where current smokers exhibited a lower understanding of CVD risk factors (*p* = 0.01).

**TABLE 4:** Knowledge of CVD risk associated with pregnancy complications according to participants' sociodemographic and clinical characteristics.

	*n*	Knowledge score mean ± SD	*F*	*p*
Age^a^				
< 25	13	15.0 ± 5.3	0.73	0. 479
25–35	190	16.1 ± 5.9		
> 35	96	16.8 ± 0.6		
Ethnicity^a^				
Australian including Australian Aboriginal/Torres Islander	175	15.6 ± 5.3	3.43	0.009
North African/Middle Eastern/Sub Saharan African	71	16.6 ± 5.3		
European/British/Irish	25	18.0 ± 6.3		
New Zealander	24	16.0 ± 5.4		
Southeast Asian/North Asian	4	24.0 ± 3.8		
Education^a^				
Primary or secondary schooling	22	13.7 ± 6.1	5.05	0.007
Certificate or diploma	97	15.4 ± 5.6		
Bachelor or postgraduate degree	180	16.9 ± 5.5		
Financial situation^a^				
Excellent	15	18.3 ± 6.7	1.00	0.406
Good	213	16.0 ± 5.3		
Fair	65	16.6 ± 5.6		
Poor	6	16.0 ± 6.9		
Marital status^a^				
Single/never married	17	14.1 ± 5.0	1.74	0.158
Married	234	16.5 ± 5.6		
De facto	40	15.2 ± 4.5		
Divorced/separated	8	17.5 ± 5.9		
BMI^a^				
Underweight	3	19.7 ± 8.9	0.67	0.566
Normal weight	136	16.5 ± 5.4		
Overweight	101	16.0 ± 5.1		
Obese	59	15.9 ± 6.0		
Smoking status				
Current smoker	12	11.3 ± 6.3	10.65	0.010
Current non‐smoker	287	16.4 ± 5.3		
Physical activity^a^				
Moderate physical activity at least 150 min per week	23	15.1 ± 7.9	2.41	0.910
Moderate physical activity less than 150 min per week	270	16.4 ± 5.2		
Little or no physical activity	6	12.0 ± 2.6		
High blood pressure^a^				
Diagnosed with high blood pressure	118	16.1 ± 5.5	1.07	0.342
Not diagnosed with high blood pressure	144	16.0 ± 5.6		
Did not know blood pressure status	37	17.4 ± 4.8		
Diabetes^a^				
Diagnosed with diabetes	70	15.7 ± 4.8	0.44	0.643
Not diagnosed with diabetes	111	16.3 ± 5.6		
Did not know blood sugar level status	118	16.4 ± 5.7		
Hyperlipidaemia^a^				
Diagnosed with hyperlipidaemia	18	15.9 ± 4.3	0.62	0.940
Not diagnosed with hyperlipidaemia	141	16.3 ± 5.4		
Did not know blood cholesterol level	140	16.2 ± 5.5		
Family history of CVD				
Positive family history	34	15.1 ± 5.4	1.63	0.202
Negative family history	265	16.4 ± 5.5		

Abbreviation: SD, standard deviation.

^a^ One‐way ANOVA; *n* = frequency.

## 4 Discussion

This study highlights a significant gap among participants' knowledge of CVD risk and symptoms. More than half of the participants were unaware of the common signs of a heart attack, aligning with findings from the Women's Heart Alliance [16]. However, the awareness of heart‐attack symptoms among participants in the current study was relatively better than that found in a survey of six European countries, where only a small proportion of women (15.4%) could recognise the most common symptoms of a heart attack apart from pain, such as dyspnea, unusual fatigue, dizziness or weakness [17].

Participants in this study showed relatively good understanding of high blood pressure as a CVD risk factor. This aligns with findings of another study conducted in the United Arab Emirates, where 62.7% of women had a good understanding of the association of high blood pressure and the CVD risk [18]. In the study conducted by Khan and Ali [18], the participants exhibited demographic characteristics comparable to those in our research. The women were aged between 18 and 55 years, and the majority (88.4%) held diploma or undergraduate degrees, closely aligning with the 92.6% observed in our study. In contrast, in a study in Chile, only 22% of women recognised it as a primary cause of CVD [19]. The demographic characteristics of the participants in the study by Varleta et al. [19] differed notably from those in our study. Their participants were aged between 30 and 75 years, whereas our study included individuals aged 18 and older. Furthermore, the majority of participants in their study (82.4%) belonged to a low or medium‐to‐low socioeconomic level, in contrast to 23.7% in our cohort. Additionally, only 17.6% of their participants had completed university or technical studies, compared to a significantly higher proportion in our study. We presume that these demographic differences may have contributed to the lower levels of knowledge regarding CVD risk factors observed in the study by Varleta et al. [19].

High blood pressure is a significant modifiable risk factor, and evidence suggests that reducing hypertension decreases mortality and CVD risk [20]. Substantial evidence suggests that reducing blood pressure in hypertensive individuals can decrease mortality and CVD risk [20]. Given that hypertension acts as an independent risk factor for CVD and can also interact with other risk factors to elevate the likelihood of CVD [21], experts widely agree that the threshold for treating hypertension in individuals at risk of CVD should be lower [22].

Women with a history of complications of pregnancy are at higher risk of developing CVD, often at a younger age than those without such history [9]. A contributing factor to low awareness among participants in this study is the perception that heart disease is not a significant health issue among women of reproductive age. Additionally, participants had low awareness of the relationship between menopause and CVD, underscoring the need to monitor cardiovascular health during midlife [23]. Educational initiatives are essential to raise awareness of CVD as the leading cause of death among women across all age groups [24].

Higher knowledge levels are linked to better health outcomes as awareness empowers individuals to adopt healthy behaviours [24]. Since many CVD risk factors, such as obesity, Type 2 diabetes, smoking and diet, are modifiable [9], educating women about these risks is crucial for prevention. Knowledge enhances health literacy, which is vital for informed decision‐making and long‐term health improvement [24].

Health beliefs also play a critical role in health behaviours [25]. The Health Belief Model suggests that perceptions of susceptibility, severity, benefits, and barriers influence individual actions [26]. Women are more likely to engage in preventive behaviours if they perceive heart disease as severe [26]. Interventions focusing on health beliefs and awareness could lead to better adherence to healthy lifestyles and lower CVD incidence among women [27].

Participants with higher education levels exhibited greater CVD knowledge, consistent with previous research. Studies in sub‐Saharan Africa [28, 29], Italy [30], and the United States [14] have found similar trends. Education positively impacts CVD awareness, healthy behaviours, working conditions and access to healthcare [27]. Conversely, lower education levels are associated with risk factors such as obesity, smoking and physical inactivity [31]. Addressing educational disparities through targeted interventions can improve health literacy and empower women to manage CVD risks effectively [27].

Most participants in the study were from higher socioeconomic backgrounds but still demonstrated gaps in CVD knowledge, particularly among those with lower education levels. This suggests even poorer knowledge among women from lower socioeconomic backgrounds due to limited health literacy [31]. Interventions targeting these groups could reduce health disparities and the overall CVD burden.

This study underscores the urgent need for educational programs targeting CVD awareness among women with pregnancy complications. Enhancing health literacy and addressing health beliefs can empower women to make informed decisions and adopt preventive measures. Since higher education levels correlate with better CVD knowledge, efforts to improve access to education and health resources are essential to mitigate disparities. The findings also emphasise the importance of targeted interventions for high‐risk and socioeconomically disadvantaged groups. Tailored educational resources and timely counselling could significantly enhance the cardiovascular health of women with a history of pregnancy complications. This approach could reduce the overall CVD burden by promoting early awareness and preventive behaviours.

### 4.1 Strengths and Limitations of the Study

This study is the first to explore CVD knowledge among women with multiple pregnancy complications, addressing a significant research gap. Previous studies focused on single complications. The survey tools were rigorously validated and pilot‐tested, ensuring reliability and internal consistency. However, the study's non‐probability sampling method introduces potential selection bias, limiting generalizability [32]. Additionally, reliance on self‐reported data may have introduced recall bias [33]. Despite these limitations, the findings provide a foundation for developing tailored interventions to enhance women's CVD awareness.

## Conflicts of Interest

The authors declare no conflicts of interest.

## Data Availability

The anonymised research data are available upon request to the authors.
